# Salicylic Acid in Pneumonia

**Published:** 1878-02

**Authors:** L. L. Silverthorn

**Affiliations:** Charleston, Ill.


*Salicylic Acid in Pneumonia.*

I was called on the morning of Aug. 24th, 1877, to see Mr. H., a vigorous young man about 25 years of age. He stated that on the previous day he had taken a good deal of exercise on horseback, had been caught in a heavy rain in the evening, and that immediately after getting into bed he had been attacked with a very severe chill. He suffered all night with general aching and pains, more especially in his right chest. I found him in the following condition: Face very much flushed, skin hot and dry, pulse 120 beats per minute, tongue coated and dry, some pain in right chest and shoulder, cough very distressing, dyspnœa, scanty expectoration of bloody mucus, bowels constipated, urine scanty and high colored, distaste for food, and thirst. Percussion elicited general dullness over the right chest; auscultation revealed general crepitation, and slight rasping over the inferior posterior region of the same side. There was also crepitation in the lower left lobe. As I had the misfortnne to break my thermometer a short time before, the temperature was not taken, but was evidently much exaggerated. The diagnosis was; an unusually severe case of pleuro-pneumonia, and the prognosis unfavorable. My first impulse was to perform venesection, but accidentally putting my hand in my pocket I discovered there a a bottle of salicylic acid; and the idea occurred to me to employ it. I had previously witnessed its effects in the febrile state, and in the inflammation of acute rheumatism. I therefore ordered 20 grains of the acid to be taken every two hours till I should see him again, the first dose to begin at 9 o'clock a. m. My plan of mixing and administering salicylic acid is as follows: It is placed in a tablespoon half full of water, and sweet spirits of nitre added, drop by drop, till the acid is moistened. The patient swallows a mouthful of sweet milk, then the acid, and finally some more milk. By this means very little complaint is made of a bad taste, or of irritation of the mouth and pharynx. At half-past 7 p. m. the cough, pain and dyspnœa were not much relieved, the bowels had not moved, and there had been no discharge of urine, but the tongue was moist, expectoration more free, sputa very bloody, free diaphoresis. He had taken five doses of the acid and no nourishment except the milk with the powders. The following powder was given at night: Calomel, grs. v.; morphia sulphate, grs. ¼; sodic bi-carbonate, grs. x.; the acid once in three hours.

Aug. 25th, 8 a. m.—Patient had perspired profusely all night and rested well after taking the powder; at 10 o'clock very little cough or expectoration, no blood, tongue moist and almost clean, lungs expanding properly, pulse natural in frequency but very feeble; much prostration. The kidneys acted but little, and this I attributed to the excessive sweating, as the blanket over him as well as the sheet beneath were fully saturated. The bowels had not moved. But one dose of the acid taken since the last visit, and no nourishment. To have castor oil ℥ i., oil of turpentine ʒ ss. at once; milk and beef tea *ad libitum;* and after an operation, grs. x of the acid every four hours.

P. M., 7½ o'clock.—Patient still sweating, but not so freely; has perspired continuously for over twenty-four hours. Bowels and kidneys have acted well, appetite improving, has taken two powders, and seems well but weak. The powders are discontinued, and aromat. spts. of ammon. ʒ ss. given every hour or two when awake, with food.

Aug. 26th, 8 a. m.—It is now just forty-eight hours since the first visit, and about fifty-eight hours since the onset of the disease. The patient has taken in all one hundred and forty grains of salicylic acid, calomel grs. v., morphia gr. ¼, sodic bi-carbonate grs. x. The patient is dressed, has taken later a good breakfast, and is quite comfortable. The ammonia was given simply as a gentle stimulant.

Is not salicylic acid an abortive in pneumonia?

L. L. SILVERTHORN, Charleston, Ill.

---

QUID SPECULUM POSSIT.—One of our most skillful practitioners recently had occasion to employ the vaginal speculum in the examination of a lady. The exploration finished, he was about to withdraw the instrument, when he felt a light touch upon his shoulder. "Excuse me, doctor," said the patient, "I have long suffered from pain in the stomach. *While you are there,* can you not tell me what is the matter?"—*Lyon Medical.*

